# Structural Transformation of Polyacrylonitrile (PAN) Fibers during Rapid Thermal Pretreatment in Nitrogen Atmosphere

**DOI:** 10.3390/polym12010063

**Published:** 2020-01-01

**Authors:** Wei Dang, Jie Liu, Xiaoxu Wang, Kaiqi Yan, Aolin Zhang, Jia Yang, Liang Chen, Jieying Liang

**Affiliations:** 1 Key Laboratory of Carbon Fiber and Functional Polymers, Ministry of Education, Beijing University of Chemical Technology, Chao-Yang District, Beijing 100029, China; buct_dangwei@163.com (W.D.); liuj@mail.buct.edu.cn (J.L.); alszhang@163.com (A.Z.); yangjcarbon@163.com (J.Y.); 2017200510@mail.buct.edu.cn (L.C.); liangjy@mail.buct.edu.cn (J.L.); 2 Changzhou Institute of Advanced Materials, Beijing University of Chemical Technology, Changzhou 213164, China; 3 State Key Laboratory of Technologies in Space Cryogenic Propellants, Technical Institute of Physics and Chemistry, Chinese Academy of Science, Haidian District, Beijing 100190, China

**Keywords:** polyacrylonitrile fibers, thermal pretreatment in nitrogen, structural transformation

## Abstract

The modification before the stabilization process could tune the exothermic behavior and the structural evolution of PAN fibers during stabilization. In this study, we demonstrate that a rapid thermal pretreatment in nitrogen can effectively mitigate the exothermic behavior of PAN fibers, such as decreasing the initial temperature, broadening the exothermal peak, and decreasing the nominal heat release during heating the fibers in air. The color of fibers has shown gradual changes from white to light yellow, yellow and brown during thermal pretreatment in nitrogen with the increase of pretreating temperature and time. The differential scanning calorimetry (DSC), Fourier Transform Infrared Spectrometer (FTIR), X-ray diffraction (XRD), and Thermogravimetric Analysis (TG) characterization have been applied to analyze the thermal properties, chemical and physical structural difference between PAN, and thermally pretreated PAN fibers. The thermal pretreatment of PAN fibers in nitrogen could induce cyclization, dehydrogenation, and cross-linking reactions, in which the cyclization play an important role on improving the cyclization index of stabilized PAN fibers. Meanwhile, the pretreatment can result in noticeable changes of the aggregation structure of PAN fibers, as indicated by the increase of crystallinity and crystalline size. These structural modifications can benefit the main cyclization reaction during stabilization and enhance the carbon yield in resultant carbon fibers. The rapid thermal pretreatment in nitrogen could increase efficiency of modification on PAN fibers, and that could save much time and energy. It is beneficial to manufacture low-cost carbon fibers and to spread the applications of carbon fibers.

## 1. Introduction

Carbon fibers have shown excellent properties such as high specific tensile strength, high specific young’s modulus, chemical resistance, corrosion resistance, and light weight [1,2,3]. Carbon fibers are widely used as reinforcement of composites, and the applications have extended from aerospace and military to modern industries, such as automobile, sports equipment, and energy [4,5]. Carbon fibers can be manufactured from a variety of polymer precursors, among which PAN is a main precursor for manufacturing carbon fibers [2,6].

During the manufacture of PAN-based carbon fibers, thermal stabilization in air atmosphere is indispensable [7]. Various reactions, such as cyclization, oxidation, dehydrogenation, and cross-linking reactions, initiated at elevated temperature during stabilization [8]. As a result, the linear PAN chains convert into cyclic structure or ladder-like structure, which could keep fibers infusible and nonflammable when carbonized at higher temperatures [9,10,11,12,13,14]. Meanwhile, thermal stabilization is also a time-consuming and energy-consuming process, which retards the commercializing and broader application of carbon fibers.

Previous reports have demonstrated that pretreatments, such as chemicals modification [15,16,17], irradiation modification [18,19,20,21,22,23], and thermal modification [24,25,26], could tune the exothermic behavior and the structural evolution of PAN fibers during stabilization. The KMnO_4_ has been a famous chemical modification on PAN fibers [15,16,17], which can induce oxidation of PAN molecules. The irradiation including UV irradiation, gamma-ray irradiation, plasma irradiation and so on causes cyclization of PAN molecules and aggregation structure of fibers [18,19,20,21,22,23]. Qin et al. [24,25] has researched that thermal treatment of PAN fiber in nitrogen and nitrogen and vapor atmosphere to improve the stabilization extent of fibers [24], and to improve the preferred orientation and mechanical properties of carbon fibers [25]. Cyclization is the main reaction during thermal treatment in nitrogen and nitrogen and vapor atmosphere. Thermal modification in air has been researched [26], and the results indicate that chemical reactions and aggregation structure changes during modification are beneficial to the structure and mechanical properties of carbon fibers. However, there are also limitations of these modification techniques, including introducing impurities [15,16,17], causing secondary damages on PAN fibers, needing professional equipment [18,19,20,21,22,23], and taking relatively long time [24,25,26]. According to our preliminary research of thermally pretreated PAN fibers in nitrogen, the thermal pretreatment in nitrogen could operate conveniently and take only tens to hundreds of seconds. Meanwhile, the equipment and the process of rapid pretreatment in nitrogen of this study are relatively simple, which can easily fit in the continuous production process of carbon fibers.

In this work, PAN fibers are pretreated in nitrogen atmosphere, and the effects of temperature and time of pretreatment on the structural evolution of PAN fibers are investigated. The thermal properties of PAN fibers are studied using differential scanning calorimetry (DSC), and the chemical and physical structure of PAN fibers is studied using FT-IR and XRD analysis.

## 2. Materials and Methods

### 2.1. Materials and Methods

The PAN precursor fibers (6000 filaments/tow, supplied by Courtalds’ LTD. Coventry, UK) were wet-spun from a copolymer of acrylonitrile/itaconic acid/methyl acrylate in a ratio of 92.8/1.2/6.0 (wt %).

The PAN precursor fibers were thermally pretreated in a tube furnace filled with nitrogen. The pretreatment parameters including temperature and time (shown in Table 1) were chosen through the results of differential scanning calorimetry (DSC) characterization (shown in Figures S1 and S2). The thermally pretreated PAN fibers were named modified PAN fibers (MFs).

### 2.2. Characterization

Differential scanning calorimetry (DSC-822, METTLER Toledo, Schwerzenbach, Switzerland) was used to decide the pretreatment temperature and time, and the test was performed as an isothermal test at several chosen temperatures for 20 min. Besides, DSC was used to investigate the exothermic properties of PAN and MFs, and the test was performed at a heating rate of 5 °C/min under air or nitrogen atmosphere, and the temperature ranged from 40 to 400 °C.

The density of fibers was measured at 23 ± 0.1 °C by using a density gradient tube filled with carbon tetrachloride and n-heptane. The element content of the fiber was characterized through an Elemental Analyzer (Thermo Fisher Scientific, Waltham, MA, USA).

A Fourier transform infrared spectrometer (FT-IR, Nicolet 8700, Thermo Fisher Scientific, Waltham, MA, USA) was used to characterize the chemical bonds of PAN and MFs. X-ray diffraction (XRD, D/max-2550 PC, RIGAKU Corporation, Tokyo, Japan) was used to characterize the microstructure of PAN and MFs, including crystallinity, average crystalline size (Lc) of hexagonal (100) crystal plane, interplanar crystal spacing (d_(100)_), and crystalline orientation through 2-theta scanning and azimuth scanning respectively, and the calculation of those were shown in Formulae (S1)–(S4).

Thermogravimetry test (STA209C, Netzsch, Bayern, Germany) for PAN and MFs was performed at a heating rate of 10 °C/min under air and nitrogen atmosphere, and the temperature ranged from 40 to 400 and 40 to 1000 °C, respectively.

## 3. Results and Discussion

**Figure 1:**
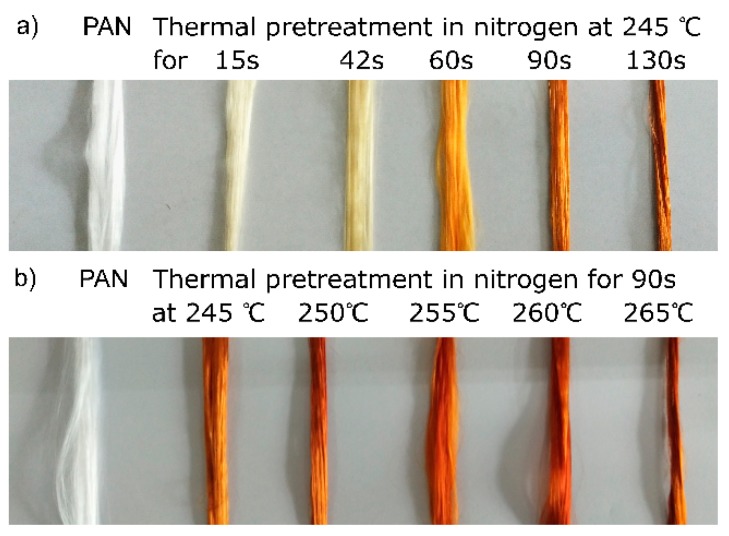
Color changes of PAN precursor fibers (white) during thermal pretreatment in nitrogen, in which (**a**) thermally pretreating PAN fibers at 245 °C for 15, 42, 60, 90, and 130 s; and (**b**) thermally pretreating PAN fibers at 245, 250, 255, 260, and 265 °C.

The color changes during thermal pretreating PAN fibers in nitrogen can exhibit the changes directly. As shown in Figure 1, it is obvious that the color of fibers gradually changes from white to light yellow, yellow, and brown during thermal pretreatment in nitrogen with the increase of temperature and time. The previous studies [7,11,13,14] have proposed that the color changes were caused by the corresponding groups generated during thermal treatment. Thus, it is proposed that reactions have occurred during thermal pretreatment in nitrogen.

**Figure 2:**
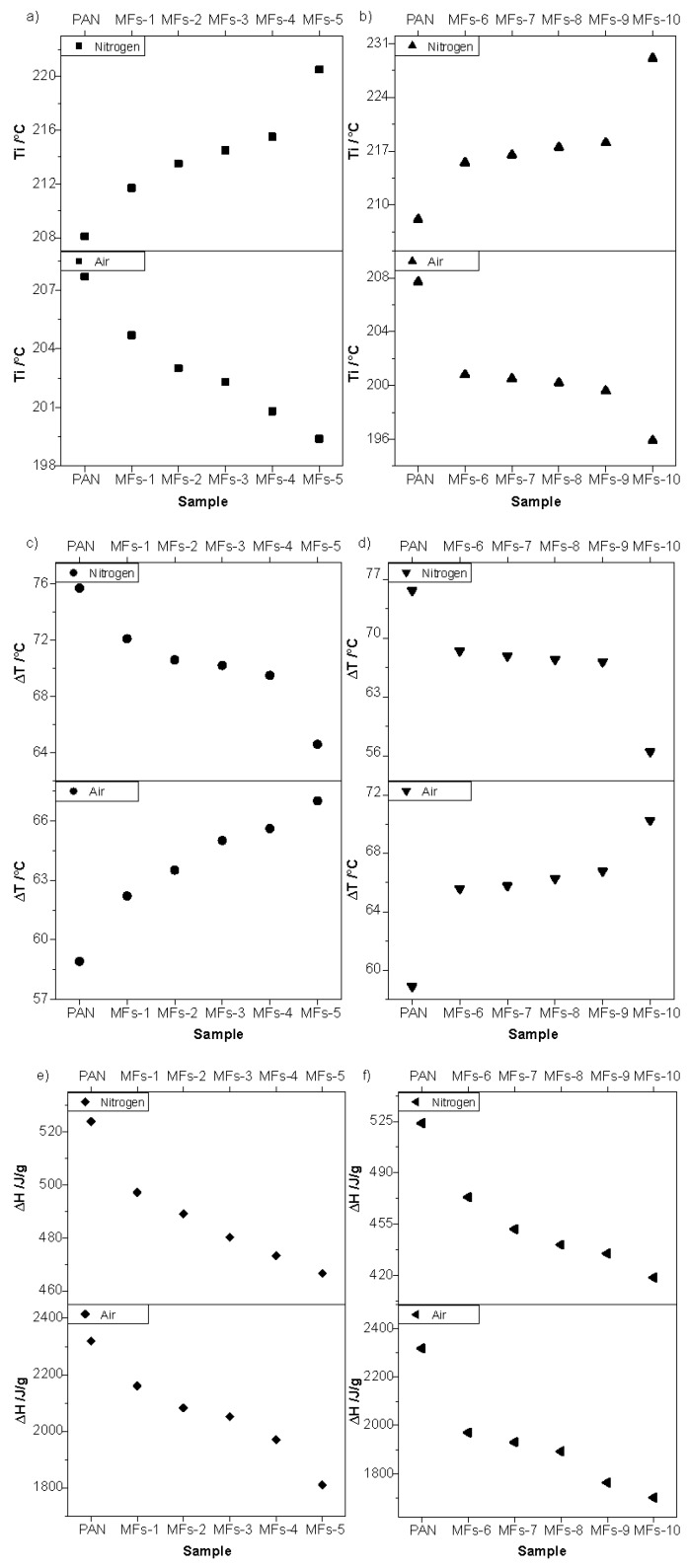
Thermal properties of PAN and modified PAN fibers (MFs) characterized through differential scanning calorimetry (DSC) in air and nitrogen, in the (**a**,**b**) initial temperature of heat releasing (T_i_), (**c**,**d**) temperature interval (ΔT) of T_i_ and T_p1_, and (**e**,**f**) nominal heat release (ΔH) of PAN and MFs.

The thermal properties of PAN fiber prior and after pretreatment were characterized through DSC (shown in Figure S3). The data of thermal properties acquired from DSC are shown in Figure 2.

The initial temperature of heat releasing (T_i_), temperature of exothermic peak (T_p_), and nominal heat release (ΔH) showed a difference between PAN and MFs. According to Figure 2a,b, T_i_ decreased in air and increased in nitrogen with the increase of pretreatment temperature and time. The T_i_ was influenced by both the chemical and physical structures of PAN fiber. The downtrend of T_i_ in air atmosphere with the increase of pretreatment temperature and time shows a lower initial temperature of pretreated fibers than that of PAN fibers. It means that the pretreated fibers need lower temperature to induce chemical and/or physical changes during thermal stabilization in air than the PAN fibers need, which implies that thermal pretreatment of PAN fibers in nitrogen may promote the reactions during thermal stabilization in air [8,9,27,28,29,30,31]. On the other hand, the uptrend of T_i_ in nitrogen atmosphere implies that the thermal pretreatment in nitrogen had induced reactions or structural changes. As a result, the activation energy of PAN fibers was increased [27,28].

The stabilization is performed in a temperature of 200–300 °C [8,9,13,29,30,31]. The maximum temperature during stabilization is typically close to the first exothermic peak temperature (T_p1_) of the DSC curve characterized in air at a heating rate of 5 °C/min. The temperature interval (ΔT) of T_i_ and T_p1_ can roughly reflect the intensity of reactions. Higher ΔT corresponding to a broader exothermic peak would result in a milder reaction. The ΔT (shown in Figure 2c,d) in nitrogen decreased with the increase of pretreatment temperature. This could be ascribed to that the active groups were reacted during thermal pretreatment in nitrogen, and the remaining part required more energy and reacted in a relatively smaller temperature range. ΔH is an evident parameter to identify the difference between samples when characterized by DSC. The ΔH of both MFs and PAN precursor fibers (shown in Figure 2e,f) were compared. Both ΔH in air and in nitrogen decreased with the increase of pretreatment temperature and time. This indicates that chemical reactions or structural changes during thermal pretreatment in nitrogen were beneficial to reactions in air (corresponding to stabilization) and nitrogen [28].

**Figure 3:**
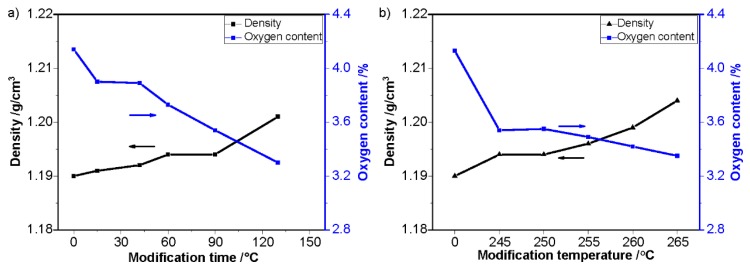
Density and oxygen content of: (**a**) PAN fibers pretreated at 245 °C with different time and (**b**) PAN fibers pretreated for 90 s under different temperatures.

To identify the chemical and physical changes during thermal pretreatment in nitrogen, several characterizations were taken. Figure 3 shows the oxygen content and density of PAN and MFs. The chemical composition of PAN can be characterized by the Elemental Analyzer, and the oxygen content is a general parameter to evaluate the chemical changes of PAN fibers during thermal treatment [10,32,33]. The density of PAN fibers and MFs is another general reflection of the chemical and physical structural evolutions of PAN fibers.

As shown in Figure 3, it was obvious that the oxygen content changed as a function of the pretreatment temperature and time. With the increase of pretreatment temperature or time, the oxygen content of PAN fibers decreased. This indicates that the original oxygen within the PAN copolymer was removed during the process of pretreatment. The densities of PAN fibers increased with the enhancement of pretreatment temperature and pretreatment time. This indicates that both the chemical and physical structural evolutions during thermal pretreatment in nitrogen led to a denser structure of PAN fibers.

**Figure 4:**
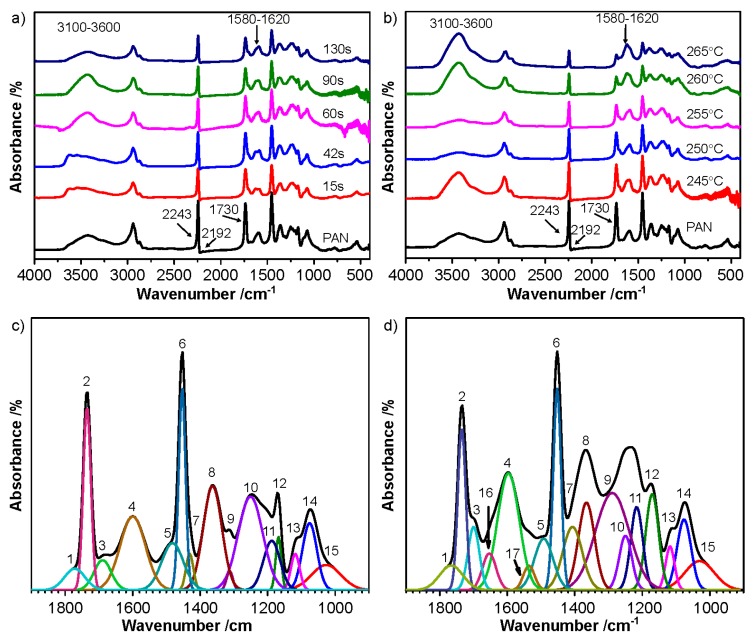
FTIR spectra of PAN and MFs pretreated (**a**) at 245 °C for different time and (**b**) at different temperatures for 90 s, and multi-peak fitting of (**c**) PAN precursor fibers and (**d**) MFs.

The chemical changes during thermal pretreatment in nitrogen were characterized through FT-IR (Figure 4), which could help to speculate the thermal-induced reactions during thermal pretreatment in nitrogen. The spectrum of PAN precursor fibers (Figure 4a,b) revealed characteristic peaks of the vibrations of nitrile group (–C≡N) at 2243 cm^−1^, carboxylic acid groups, including C=O group at 1730 cm^−1^, C–O and O–H groups at 1200 cm^−1^, and O–H group at 3100–3600 cm^−1^, respectively [9,34,35,36,37]. It is reasonable to speculate that cyclization is the main reaction during thermal pretreatment in nitrogen because there is limited oxygen in the atmosphere. The cyclization converts –C≡N groups to cyclic –C=N– and –C–N– groups, as revealed by the absorbance decrease at 2243 cm^−1^ and the appearance of broad peaks at 1580–1620 cm^−1^. For the broad peak at 1580–1620 cm^−1^, a multi-peak fitting was used to separate wavenumbers corresponding to characteristic peaks. The multi-peaks fitting ranging from 1000–1800 cm^−1^ (shown in Figure 4c,d) were also overlapping, meanwhile, the position and corresponding vibration model of peaks in typical FTIR analysis are listed in Table 2. Comparing the FTIR spectra of PAN precursor fibers and MFs, the change of absorbance could indicate the chemical structure evolution during thermal pretreatment in nitrogen. Two new peaks, 1654 cm^−1^ (C=C and C=N) [35,38,39,40] and 1535 cm^−1^ (C–N and N–H of amide) [40], appeared during thermal pretreatment in nitrogen, which were also associated with the decrease of absorbance in 2243 cm^−1^ (–C≡N). This indicates that the cyclization had occurred during thermal pretreatment in nitrogen.

**Figure 5:**
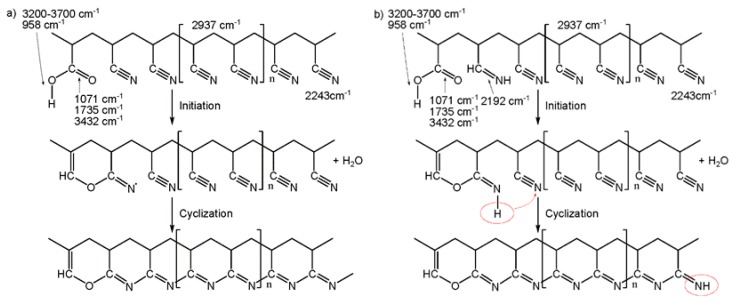
Suggested mechanism of thermal pretreatment in nitrogen, (**a**) the standard PAN copolymer of AN/IA/MA and (**b**) the PAN copolymer with hydrolysate of nitrile groups.

Based on the chemical evidence provided by FT-IR analysis, the suggested mechanism of thermally pretreated PAN fibers in nitrogen is shown in Figure 5. The cyclization is the main reaction during thermal pretreatment in nitrogen process. However, there are also dehydrogenation and chain scission reactions occurring at the same time. These reactions result in the removal of oxygen from the PAN copolymer chains [9,10,24,37,39].

According to previous studies, the cyclization plays an important role on forming ladder structure of stabilized PAN fiber, and the ladder structure could keep the fibers nonflammable and infusible [10,11,12]. The cyclization extent during thermal pretreatment in nitrogen can be evaluated by relative cyclization index (RCI, the calculation of RCI is shown in Supplementary Information, Formula S5), as well as evaluating that of stabilized PAN fibers [10,11,12,24]. The RCI of MFs in this study is over 20%. Comparing with the traditional stabilization, the RCI of stabilized fibers is about 60%–80% [8,13,24,29]. So, thermal pretreating PAN fibers in nitrogen could improve the cyclization index of fibers.

The transformation of chemical structures typically associates with the change of aggregation structures. The crystalline parameters including crystallinity, crystalline size (L_c_), interplanar crystal spacing (d_(100)_), and crystallite orientation were characterized through XRD (Figure S4). The crystalline parameters, which can reflect the aggregation structure changes during thermal pretreatment in nitrogen, are shown in Table 3.

It was evident that the crystallinity and crystalline size (L_c_) of MFs were higher than those of PAN precursor fibers, and the interplanar crystal spacing (d_(100)_) and orientation of MFs were basically unchanged compared with those of PAN precursor fibers. The crystallinity of MFs increased with the increase of pretreatment time, whereas the increase terminated at the temperature of 250 °C and then decreased with enhancing the pretreatment temperature. This revealed that the crystallinity of MFs was improved during thermal pretreatment in nitrogen, and the effect of pretreatment temperature showed greater impact than pretreatment time. The L_c_ of MFs increased first then decreased with increasing pretreatment temperature or time. Since the L_c_ could reflect the length of ladder structure of MFs, the larger L_c_ indicates that MFs had formed a ladder structure during thermal pretreatment in nitrogen. The thermally stable ladder structure is beneficial to carbonization [38]. The interplanar crystal spacing (d_(100)_) shows no difference between PAN precursor fibers and MFs, which indicates that thermal pretreatment in nitrogen did not induce changes on the basic crystalline. The orientation shows no difference between MFs and PAN precursor fibers. This could be ascribed to that the cyclization induced shrinkage played an important role on the amorphous region, which transforms the irregular structure of the amorphous region to a regular structure of the crystalline, and the stack lattice is improved. The increasing of crystallinity and crystalline size (L_c_) of PAN precursor fibers may benefit the mechanical properties of carbon fibers [41,42].

**Figure 6:**
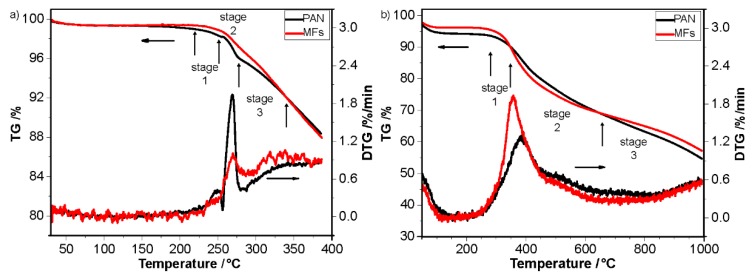
TG-DTG of PAN and MFs, (**a**) at temperatures ranging from 40 to 400 °C in air, and (**b**) at temperatures ranging from 40 to 1000 °C in nitrogen.

Figure 6 shows the TG-DTG of PAN fibers and MFs in air and nitrogen atmosphere, which can help to interpret the effects of thermal pretreatment in nitrogen on the stabilization and carbonization [27,28].

The mass loss means the interpolation of mass percentage of different temperature at TG curves. The temperature of stabilization and low-temperature carbonization typically ranges from 200 to 300 °C and 400 to 800 °C, respectively [9,43,44]. In Figure 6a, the initial mass loss temperature of both PAN and MFs was about 215 °C, and the mass loss of MFs was lower than that of PAN fibers at the temperature ranging from 215 to 317 °C [27,45]. In the first stage, the mass loss of both PAN fibers and MFs was little, which might be corresponding to the little initial reactions’ occurrence. In the second stage, the mass loss shows difference between PAN fibers and MFs. The mass loss of PAN fibers shows a small horizontal transition and then increased with the increase of temperature, while that of MFs shows a steady increase in this stage. It indicates that the reactions of MFs in the second stage were steadier than that of PAN fibers. In the third stage, the mass loss of both PAN fibers and MFs was steady and higher than in the first and second stage. When the temperature increased over 317 °C, the mass loss of MFs was higher than that of PAN fibers, which means that the reactions in this stage was steady and at a high reaction rate. It indicates that thermal pretreatment in nitrogen could benefit to control the reactions and promote the reaction at a high temperature of the stabilization process [9]. In Figure 6b, the TG curves of both PAN fibers and MFs in nitrogen atmosphere could also be divided into three stages. In the first stage, the mass loss of both PAN fibers and MFs was little, and might be caused by the initial cyclization. In the second stage, the mass loss of MFs was higher than that of PAN fibers, and both were steady. It means that the reactions of MFs was at a higher rate than the PAN fibers in the temperature range from 350 to 650 °C, which indicates that thermal pretreatment in nitrogen could promote the carbonization process. In the third stage when the temperature was higher than 650 °C, the mass loss of MFs was lower than that of PAN fibers, which means that thermal pretreatment in nitrogen could benefit the carbon yield at the carbonization process [43,44]. As a result, thermal pretreatment in nitrogen could moderate the stabilization process and facilitate the carbonization process, and benefit the carbon yield at the carbonization process.

## 4. Conclusions

The rapid thermal pretreatment in nitrogen could induce a cyclization reaction in PAN fibers, which is beneficial to the main cyclization reaction during the subsequent stabilization process. Meanwhile, unlike previous reports on long-time pretreated PAN fibers with deteriorated aggregation structures, the rapid pretreatment could increase the crystallinity and crystalline size of PAN precursor fibers at a proper temperature range. These modified chemical and physical structures in pretreated PAN fiber could facilitate the carbonization reaction and give rise to higher carbon yield in resultant carbon fibers. The rapid thermal pretreatment in nitrogen is convenient and needs tens to hundreds of seconds, which could increase the efficiency of modification on PAN fibers, and save much time and energy of the production. It is beneficial to manufacture low-cost carbon fibers and spread the applications of carbon fibers [45].

## Figures and Tables

**Table 1:** Parameters of thermal pretreatment of PAN precursor fibers.

Sample	Temperature/°C	Time/s	Sample	Temperature/°C	Time/s
MFs-1	245	15	MFs-6	245	90
MFs-2	245	42	MFs-7	250	90
MFs-3	245	60	MFs-8	255	90
MFs-4	245	90	MFs-9	260	90
MFs-5	245	130	MFs-10	265	90

**Table 2:** Position and corresponding vibration model of peaks in multi-peak fitting of FTIR spectra.

Designation of Peaks	Position of the Peaks (cm^−1^)	Corresponding Vibration Modes	References
1	1773	ν_C = O_	[35]
2	1735		
3	1690	ν_C = O_ in dimer COOH	[35]
4	1600	ν_C = C_ + ν_C = N_ + δ_N-H_	[35,38]
5	1481	ν_C = C_ of ring	[36]
6	1452	δ_C-H_ in CH_2_	[35,36]
7	1430	δ_O-H_ and ω_C-H_	[35]
8	1362		
9	1312		
10	1250	ν_asC-O-C_ in ether	[36]
11	1189	ν_C-O_	[38]
12	1168	ν_C-C_ + ν_C-N_ + ν_C-O_ + δ_O-H_ of COOH	[22,35]
13	1117		
14	1074	ν_sC-O-C_ in ether	[22,35]
15	1027	ν_C-N_ in aliphatic amine	[35,36]
16	1654	ν_C = O_ in acridoneν_C = C_ and ν_C = N_	[24,35]
17	1535	ν_C-N_ and ν_N-H_ of amide	[24,35]

**Table 3:** Crystalline parameters of PAN and MFs characterized through XRD.

Sample	Crystallinity/%	L_c_/nm	d_(100)_/Å	Orientation/%
PAN	61.9	6.58	5.29	85.3
MFs-1	63.3	7.42	5.28	86.3
MFs-2	66.1	7.69	5.30	86.5
MFs-3	67.1	7.92	5.32	86.5
MFs-4	67.1	7.90	5.31	85.5
MFs-5	67.7	7.45	5.30	86.7
MFs-6	67.1	7.90	5.35	85.5
MFs-7	68.1	8.04	5.31	86.4
MFs-8	67.7	7.96	5.28	86.6
MFs-9	67.6	7.69	5.29	86.6
MFs-10	67.0	7.49	5.30	85.9

## Supplementary Materials

The following are available online at https://www.mdpi.com/2073-4360/12/1/63/s1, Figure S1: DSC curves of PAN precursor fibers in air and nitrogen atmosphere, Figure S2: Isothermal DSC curves of PAN precursor fibers, (**a**) in air and (**b**) in nitrogen atmosphere, Figure S3: DSC curves of PAN precursor fibers and MFs pretreated for different duration time under (**a**) air and (**b**) nitrogen atmosphere, and DSC curves of PAN precursor fibers and MFs modifier at different temperature under (**c**) air and (**d**) nitrogen atmosphere, Figure S4: XRD spectra of PAN precursor fibers and MFs, (**a**) 2-theta scanning and (**b**) azimuth scanning of MFs pretreated at 245 °C for different time, (**c**) 2-theta scanning and (**d**) azimuth scanning of MFs pretreated at different temperature for 90 s.
