# Supplementary material for: Structural Transformation of Polyacrylonitrile (PAN) Fibers during Rapid Thermal Pretreatment in Nitrogen Atmosphere
Source: Polymers (Basel). 2020 Jan 1;12(1):63. doi: 10.3390/polym12010063 (PMC7023665; doi:10.3390/polym12010063)
Supplement: Supplementary file 1 [file polymers-12-00063-s001.pdf]

**Supplementary information**

# Structural transformation of polyacrylonitrile (PAN) fibers during rapid thermal pretreatment under nitrogen atmosphere

**Wei Dang [1], Jie Liu [1,2], Xiaoxu Wang [1,2,*], Kaiqi Yan [3,*], Aolin Zhang [1], Jia Yang [1], Liang Chen [1], and Jieying Liang [1]**

[1] Key Laboratory of Carbon Fiber and Functional Polymers, Ministry of Education, Beijing University of Chemical Technology, Chao-Yang District, Beijing 100029, China.

[2] Changzhou Institute of Advanced Materials, Beijing University of Chemical Technology, Changzhou, Jiangsu 213164, China.

[3] State Key Laboratory of Technologies in Space Cryogenic Propellants, Technical Institute of Physics and Chemistry, Chinese Academy of Science, Haidian District, Beijing 100190, China.

* Correspondence:
Tel: +86 10 64438724; wangxiaoxu@mail.buct.edu.cn (X.W.)
Tel: +86 10 82543690; yankaiqi@mail.ipc.ac.cn (K.Y.)

**DSC test of PAN precursor fibers for choosing pretreatment temperature and time.**

To decide the pretreatment temperature, the DSC test has been performed at a heating rate of 5 °C/min under air and nitrogen atmosphere, and the temperature ranges from 180 to 400 °C (shown in Figure S1). To decide the duration time, the DSC test bas been performed at several chosen temperature for isothermal test (shown in Figure S2).

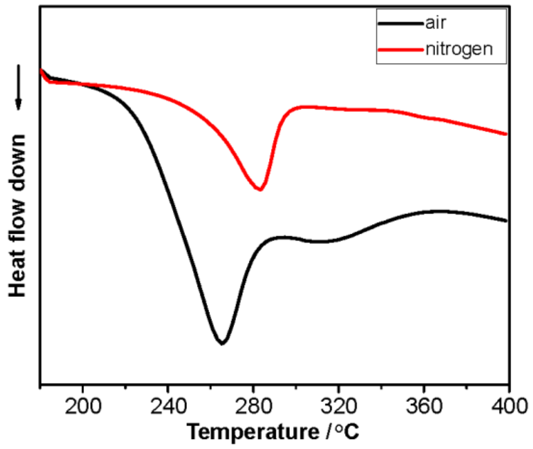


Figure S1 DSC curves of PAN precursor fibers in air and nitrogen atmosphere

When choosing the pretreatment temperature, the relation between reactions and temperature should take into consideration. If the pretreatment temperature is too low, the reactions will not occur or occur at a low efficiency; if the pretreatment temperature is too high, the reactions will be too rapid and acute to be controlled. For the pretreatment is performed in nitrogen atmosphere, so the temperature lower limit is the initial exothermic temperature of DSC characterization in nitrogen. Meanwhile, the pretreated PAN fibers would be stabilized in atmosphere in carbon fibers manufacture, so the temperature upper limit is the first exothermic peak temperature of DSC characterization in air.

The previous reports have proposed that the cyclization had induction period when heating in nitrogen. When the temperature reaches to the reacting temperature, the DSC curves shows no heat release; but when heating the PAN precursor fibers for several minutes, the resulted fibers shows difference compared with PAN precursor fibers when characterized through FTIR. The several minutes has been named induction period of cyclization. But in this work, the DSC curves (shown in Figure S2) shows obvious heat release both in air and in nitrogen atmosphere, and the heat release reaches to the exothermic peak in tens to hundreds of seconds. On the other word, no obvious induction period appears. So, we choose relatively short time for the thermal pretreatment in nitrogen.

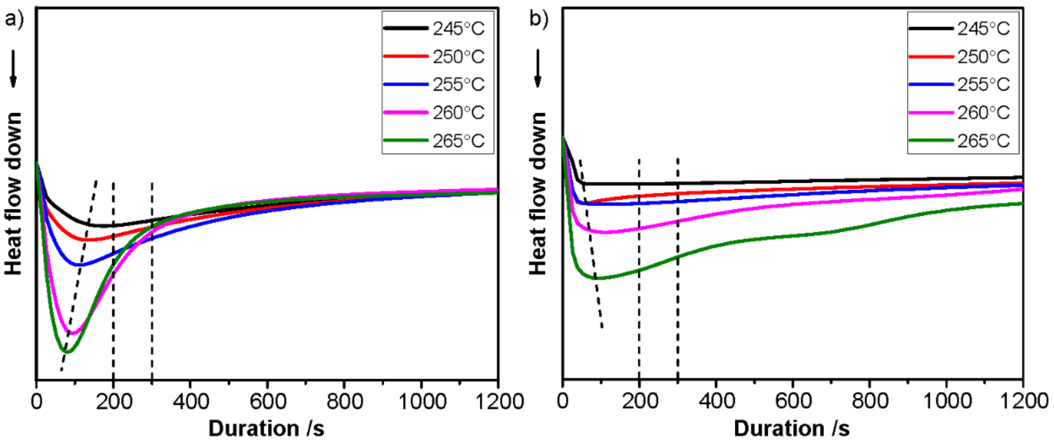


Figure S2 Isothermal DSC curves of PAN precursor fibers, (a) in air and (b) in nitrogen atmosphere

**Thermal properties characterized through DSC at a heating rate of 5 °C/min.**

The DSC curves of PAN precursor fibers and MFs are shown in Figure S3.

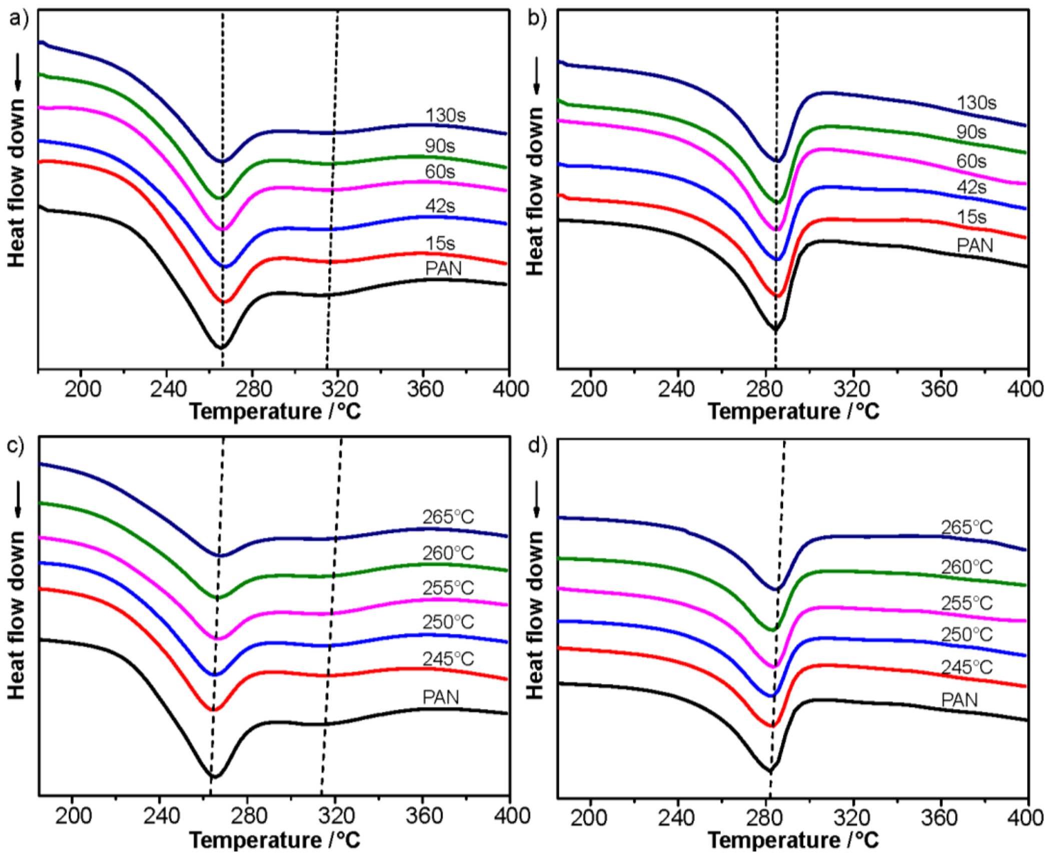


Figure S3 DSC curves of PAN precursor fibers and MFs pretreated for different duration time under (a) air and (b) nitrogen atmosphere, and DSC curves of PAN precursor fibers and MFs modifier at different temperature under (c) air and (d) nitrogen atmosphere

**XRD characterization of PAN and pretreated PAN fibers.**

The XRD characterization is performed by both 2-Theta and azimuth scanning to characterize the crystalline and orientation respectively, shown in Figure S4.

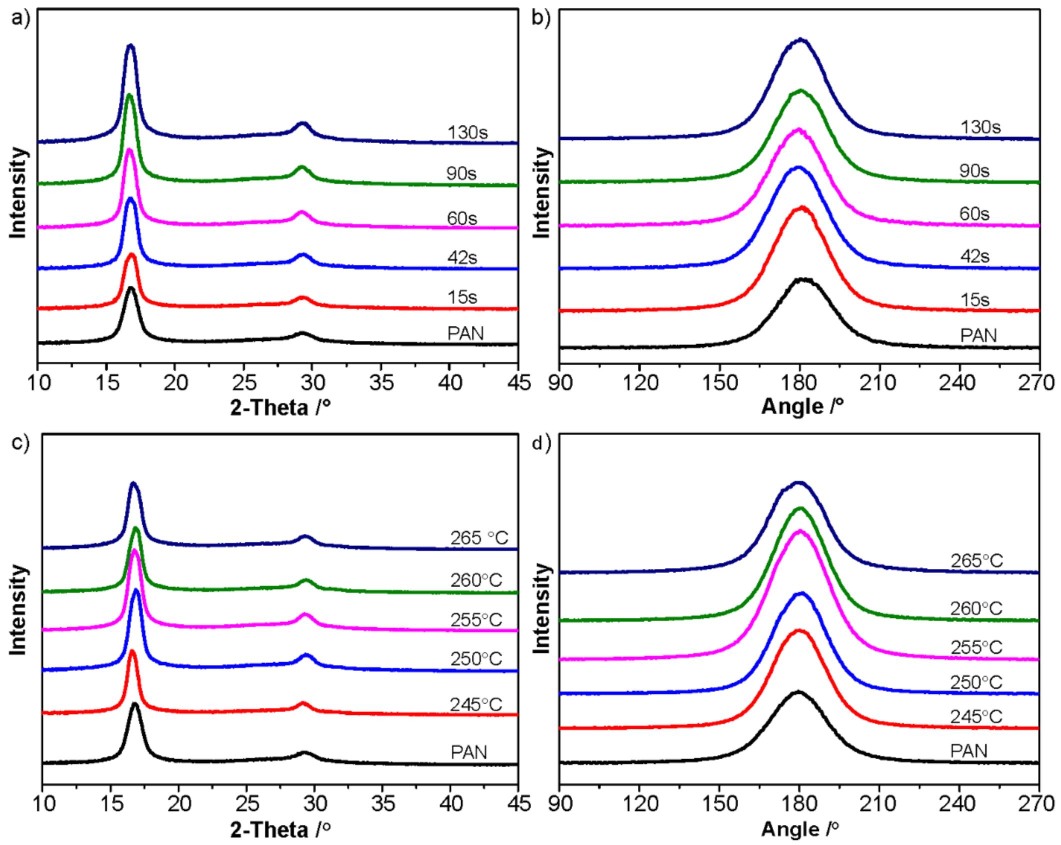


Figure S4 XRD spectra of PAN precursor fibers and MFs, (a) 2-theta scanning and (b) azimuth scanning of MFs pretreated at 245 °C for different time, (c) 2-theta scanning and (d) azimuth scanning of MFs pretreated at different temperature for 90s

The crystallinity is calculated through the formula:

$$C\% = \frac{A_c}{A_c + A_a} \times 100\% \quad \text{(S1)}$$

in which, Ac and Aa are the integral area of crystalline and amorphous region, respectively.

The Lc and $d_{(100)}$ are calculated through Scherrer equation:

$$L_c = \frac{k\lambda}{\beta\ cos\theta} \quad \text{(S2)}$$

$$2dsin\theta = n\lambda \quad \text{(S3)}$$

in which, *k* is the apparatus constant (0.89); λ = 0.15418 nm is the wavelength of the X-rays; β is the full width at half maximum in radians at 2θ = 16.9°; d is the interplanar crystal spacing of (100) crystal plane.

The crystalline orientation (φ) is calculated through formula:

$$\varphi = \frac{180-H}{180} \times 100\% \quad \text{(S4)}$$

in which, H is the full width at half the maximum intensity at 2θ = 16.9° diffraction. In the calculation, the MDI Jade (v6.0) and PeakFit (v4.12) software have been used for multi-peak fitting.

**FTIR characterization of PAN fibers and MFs.**

The cyclization extent of MFs during thermal pretreatment in nitrogen is evaluated through relative cyclization index (RCI), characterized through FTIR, and calculated through Formula S5:

$$RCI = \frac{I_{C=N}}{I_{C\equiv N} + I_{C=N}} \times 100\% \qquad (5)$$

in which, $I_{C\equiv N}$ and $I_{C=N}$ are intensity of C≡N and C=N in FTIR spectrum. The intensity could be expressed through two methods: the area of the peak and the height of the peak. In this study, the intensity is expressed through the height of the peak.

**SEM characterization of PAN and MFs**

The SEM characterization of PAN and pretreated fibers (modified PAN fibers, MFs) has been done to analyze the surface changes during thermal pretreatment in nitrogen. The SEM images are shown in Figure S5.

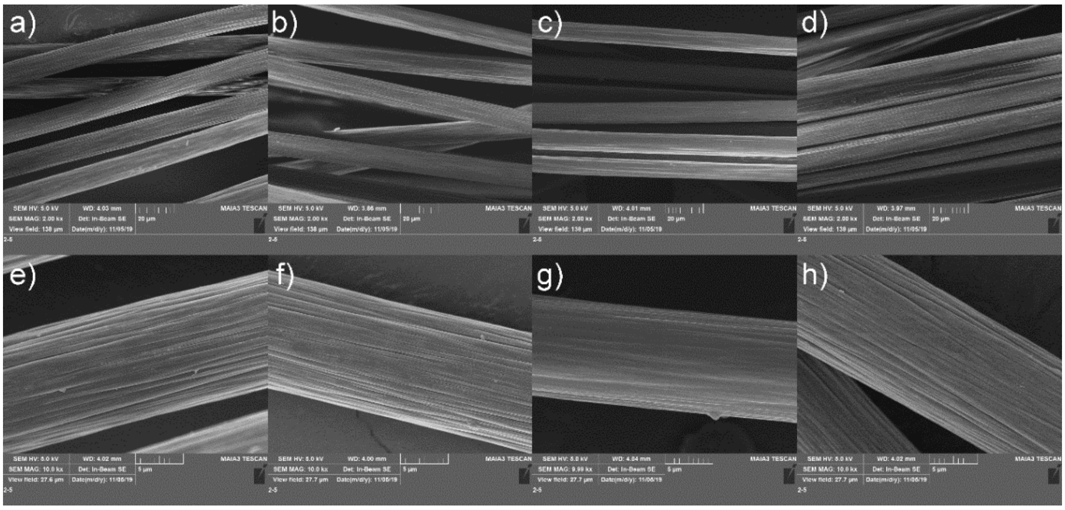


Figure S5 Original SEM images of PAN and MFs, in which PAN fibers a) and e) without thermal pretreatment in nitrogen; b) and f) with thermal pretreatment in nitrogen at 245 °C for 90s (MFs 4/6); c) and g) with thermal pretreatment in nitrogen in nitrogen at 245 °C for 130s (MFs 5); d) and e) with thermal pretreatment in nitrogen at 265 °C for 90s (MFs 10).

In Figure S5, there is no obvious difference among PAN fibers and pretreated PAN fibers. The surface texture of wet-spun PAN fibers shows no obvious changes after thermal pretreatment. Only the diameter of fibers shows tiny decrease after pretreatment, which may be caused by the strain induced by cyclization during pretreatment. So we consider that thermal pretreatment in nitrogen does not influence the surface of PAN fibers.
